# Supplementary material for: Causal Relationship between Aging and Anorexia Nervosa: A White-Matter-Microstructure-Mediated Mendelian Randomization Analysis
Source: Biomedicines. 2024 Aug 16;12(8):1874. doi: 10.3390/biomedicines12081874 (PMC11351342; doi:10.3390/biomedicines12081874)
Supplement: Supplementary file 1 [file biomedicines-12-01874-s001.zip › Supplementary Figure/Figure S8/Leaveoneout plot of MR analysis for TL to ubm-b-1775.pdf]

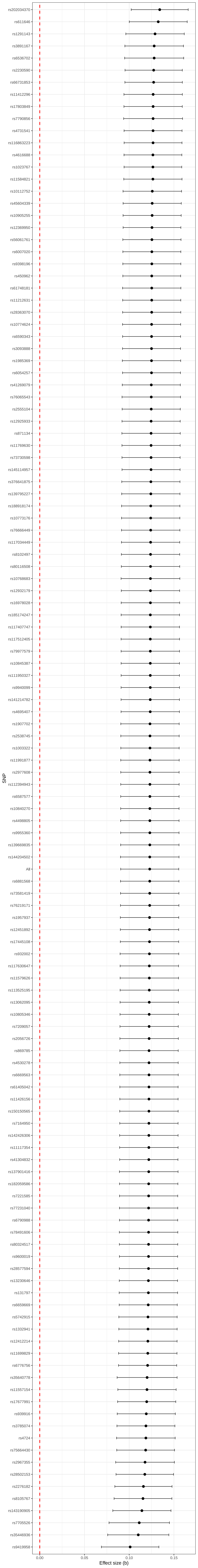
rs202034370
rs611646
rs1291143
rs3891167
rs6536702
rs2230590
rs66731853
rs11412296
rs17803849
rs7790856
rs4731541
rs116863223
rs4616688
rs1023767
rs11584821
rs10112752
rs45604339
rs10905255
rs12369950
rs56061761
rs6007020
rs9398196
rs450962
rs61748181
rs11212631
rs28363070
rs10774624
rs6590343
rs3093888
rs1985369
rs6054257
rs41269079
rs76065543
rs2555104
rs12925933
rs871134
rs11769630
rs73730598
rs145114957
rs376641875
rs139795227
rs188918174
rs10773176
rs76666449
rs117034449
rs8102497
rs80116508
rs10768683
rs12932179
rs16978028
rs185174247
rs117407747
rs117512405
rs79977579
rs10845387
rs111950327
rs9940099
rs141214782
rs4695407
rs1907702
rs2538745
rs1003322
rs11991877
rs2977608
rs112394943
rs6587577
rs10840270
rs4498805
rs9955360
rs139669835
rs144204502
All
rs6881568
rs73581419
rs76219171
rs1957937
rs12451892
rs17445108
rs932002
rs117630647
rs11579626
rs113525195
rs13062095
rs10805346
rs7209057
rs2056726
rs869785
rs4530278
rs6669563
rs61405042
rs11426156
rs150150565
rs7164950
rs142426306
rs11117354
rs41304832
rs137901416
rs182059586
rs7221585
rs77231040
rs6790988
rs78491606
rs80324517
rs9600019
rs28577594
rs13230646
rs131797
rs6659669
rs5742915
rs1332941
rs12412214
rs11699829
rs6776756
rs35640778
rs11557154
rs17677991
rs939916
rs3785074
rs4724
rs75664430
rs2967355
rs28502153
rs2276182
rs8105767
rs143190905
rs7705526
rs35446936
rs9419958
SNP
0.00
0.05
0.10
0.15
Effect size (b)
